# Hsp90 Inhibition Reduces TLR5 Surface Expression and NF-*κ*B Activation in Human Myeloid Leukemia THP-1 Cells

**DOI:** 10.1155/2018/4319369

**Published:** 2018-02-14

**Authors:** Bon Hyang Na, Thi Xoan Hoang, Jae Young Kim

**Affiliations:** Department of Life Science, Gachon University, Seongnam, Gyeonggi-do 461-701, Republic of Korea

## Abstract

Tumors highly express active heat shock protein 90 (Hsp90), which is involved in tumor survival and progression. Enhanced Toll-like receptor (TLR) 5 expression and signaling were reported to be associated with acute myeloid leukemia. In the present study, we investigated the possible modulatory effects of Hsp90 inhibitors on TLR5 expression and signaling in the human myeloid leukemia cell line THP-1. Cells were pretreated with various concentrations of the Hsp90 inhibitor geldanamycin (GA) or the Hsp70 inhibitor VER155008, followed by stimulation with bacterial flagellin. Flagellin-induced nuclear factor-*κ*B (NF-*κ*B) activation was significantly reduced by treatment with GA or VER155008. To elucidate the underlying mechanism of this effect, mRNA and cell surface expression of TLR5 was examined. TLR5 mRNA expression was enhanced by both GA and VER155008, whereas cell surface expression of TLR5 was reduced by three different Hsp90 inhibitors, including GA, 17-(allylamino)-17-demethoxygeldanamycin, and radicicol, and an Hsp70 inhibitor. The inhibitory effect of Hsp90 inhibitors was much higher than that of Hsp70 inhibitor. Our results suggest that Hsp90 inhibitors suppress TLR5 surface expression and activation of NF-*κ*B in THP-1 cells in response to TLR5 ligand, and these inhibitory effects may be associated with the possible mechanisms by which Hsp90 inhibitors suppress myeloid leukemia.

## 1. Introduction

Heat shock proteins (Hsp), including Hsp90, are highly abundant and ubiquitous molecular chaperones that help maintain the stability of proteins and target the degradation of unfolded proteins when cells are exposed to heat shock or other kinds of stress [1–3]. In addition to its role in protein folding, Hsp90 contributes to various cellular processes such as signal transduction and transportation of target protein [4, 5]. Hsp90 is widely expressed and exists as a latent and uncomplexed form in eukaryotic normal cells. In contrast, tumors highly express active Hsp90 as a complex with oncogenic client proteins [6]. Hsp90 exhibits antiapoptotic functions and stabilizes many kinases involved in cancer-cell signaling [7–9]. Since Hsp90 inhibition can influence many proteins and block signaling pathways involved in tumor survival and progression [10, 11], Hsp90 inhibitors have been considered as good antitumor agents, and some inhibitors have been investigated in clinical studies [12, 13].

Toll-like receptors (TLRs) are germline-encoded pattern-recognition receptors that play a crucial role in the first line of host defense by sensing pathogen-associated molecular patterns (PAMPs) [14]. Once TLRs recognize PAMPs, they serve as an important link between innate and adaptive immune responses by triggering the production of proinflammatory cytokines through the nuclear factor-*κ*B (NF-*κ*B) pathway, which is an essential signal transduction cascade in inflammatory responses [15]. However, while TLR signaling is important for normal immune response, enhanced or aberrant TLR activation is associated with ineffective hematopoiesis and hematopoietic malignancy [16, 17]. More notably, enhanced expression of the components of the TLR signaling pathway is associated with myelodysplastic syndromes [18–23]. Two studies have demonstrated that enhanced TLR5 expression and signaling are associated with acute myeloid leukemia [24] and multiple myeloma [25].

The purpose of this study was to investigate the possible modulatory effects of Hsp90 inhibitors on TLR5 expression and NF-*κ*B activation in human myeloid leukemia THP-1 cells.

## 2. Materials and Methods

### 2.1. Cell Culture

Human monocytic THP-1 cells (Korean cell line bank, Seoul, Korea) were grown in RPMI-1640 media (Welgene Inc., Daegu, Korea) with 10 mM HEPES buffer (Invitrogen Corp., Gibco BRL, MD, USA) and *β*-mercaptoethanol (Invitrogen Corp.) supplemented with 10% heat-inactivated fetal bovine serum and 1% antibiotic-antimycotic (Invitrogen Corp.). The cells were maintained at 37°C in a 5% CO_2_ humidified incubator.

### 2.2. NF-*κ*B/AP-1 Activation Reporter Assay

NF-*κ*B activation was measured using THP-1-Xblue cells, which are reporter cells expressing the embryonic alkaline phosphatase gene under the control of a promoter inducible by the transcription factors, NF-*κ*B and AP-1. Cells were seeded in 24-well culture plates at 0.8 × 10^6^ cells/well or 96-well culture plates at 2 × 10^5^ cells/well and preincubated for 2 h with or without the Hsp90 inhibitor geldanamycin (GA) or the Hsp70 inhibitor VER155008, followed by stimulation with 10 and 100 ng/ml of flagellin for 24 h. A 20 *μ*l aliquot of the supernatant was collected from flagellin-stimulated cultures and then added to 180 *μ*l of QUANTI-Blue alkaline phosphatase detection medium (InvivoGen, USA) for color development at 37°C. After 2-h color development, absorbance was measured at 630 nm using an ELISA microplate reader (*μ*-Quant; Bio-Tek Instruments, Winooski, USA).

### 2.3. Treatment of Cells with Inhibitors

The Hsp90 inhibitor, GA, was purchased from Sigma-Aldrich (St. Louis, MO, USA). Cells were treated with different concentrations of GA (5, 10, 20, 50, 100, 200, and 500 nM) for 24 h. 17-(Allylamino)-17-demethoxygeldanamycin (17-AAG) and radicicol were purchased from Cayman Chemical (Ann Arbor, MI, USA). Cells were treated with different concentrations of each Hsp90 inhibitor, according to specific treatments. The Hsp70 inhibitor, VER155008, was obtained from Sigma-Aldrich.

### 2.4. Flow Cytometry

To determine the surface expression of TLR5, cells were incubated with a purified anti-TLR5 antibody (MA5-16236, Thermo Fisher Scientific, Waltham, MA, USA) for 30 min and then incubated with phycoerythrin-conjugated secondary antibody (goat IgG; CLCC35004; Cedarlane Lab, Burlington, Ontario, Canada) at 4°C for 30 min. After washing with phosphate buffered saline (PBS), cells were resuspended in PBS before analysis on a Cytomics FC500 MLP (Beckman Coulter Inc., Fullerton, CA, USA). To determine intracellular TLR5 expression, cells were incubated with 50 *μ*l of fixation buffer for 30 min at room temperature followed by 30 min incubated with 50 *μ*l of 1x permeabilization buffer. Cells were then stained with a purified anti-TLR5 antibody (19D759.2, Novus Biologicals, Littleton, Colorado, USA) for 30 min and then incubated with phycoerythrin-conjugated secondary antibody (goat IgG; CLCC35004; Cedarlane Lab) at 4°C for 30 min. After washing with PBS, cells were resuspended in PBS before analysis on a Cytomics FC500 MLP (Beckman Coulter Inc.).

### 2.5. Quantitative Real-Time Polymerase Chain Reaction (PCR)

In order to analyze TLR5 expression, total RNA was extracted with the Qiagen RNeasy kit (Qiagen) according to the manufacturer's instructions. RNA concentrations were determined with a MaestroNano Micro-Volume Spectrophotometer (Maestrogen, Las Vegas, NV, USA). cDNA was synthesized from 2 *μ*g of total RNA using Hyperscript RT master mix (GeneAll, Seoul, Korea) using an Oligo (dT) primer (Invitrogen) at 42°C for 1 h.

Quantitative real-time PCR was performed on the rotor-gene system (Qiagen) using the Platinum SYBR Green qPCR SuperMix-UDG (Invitrogen). PCR amplification was performed using the following primer sets: TLR5 5′-ccttacagcgaacctcatccac-3′, 5′-tccactacaggaggagaagcga-3′, *β*-actin 5′-gacttccctactctcatctgct-3′, 5′-cttattctaggggcagagggt-3′. Sample normalization was performed using the human *β*-actin gene as an endogenous control. For each sample, the relative abundance of target mRNA was calculated from the C_Δt_ values of the target and endogenous *β*-actin reference genes using the 2^−ΔΔ^ cycle threshold (Ct) method.

### 2.6. Western Blot Analysis

For NF-*κ*B activation, nuclear and cytoplasmic proteins were separated using NucBuster Protein Extraction Kit (Novagen, Rockland, USA). Proteins were separated on 8% SDS-polyacrylamide gels and transferred onto PVDF membranes. After blocking with 5% BSA solution, the membranes were incubated overnight with primary antibody against NF-*κ*B p65 (sc-372, Santa Cruz) at 4°C. Subsequently, the membrane was washed using TBS with Tween (TBST) and then incubated with secondary antibody solutions at room temperature for 2 hr. Blots were again washed with TBST and then developed with the ECL Plus Western Blotting Detection System. *β*-Actin and lamin B were used as positive control for the cytoplasmic and nuclear proteins, respectively.

For TLR5, THP-1 cells were treated with GA at different concentrations (20, 50, 100 200, and 500 nM). The same procedure of Western blot analysis as described above was performed with an anti-TLR5 antibody (19D759.2, Novus Biologicals).

### 2.7. Statistical Analysis

Data were analyzed by one-way analysis of variance (ANOVA) followed by post hoc comparisons either with the Tukey HSD (honestly significant difference test) for groups of data with equal variances or with the Games–Howell test for unequal variances using SPSS 12.0 for Windows. All experiments were performed in duplicate. Data from two independent experiments with different batches were pooled for statistical analysis. Values are expressed as means ± standard deviation (SD). Statistical significance was defined as *P* < 0.05.

## 3. Results

### 3.1. Hsp90 and 70 Inhibitors Decrease Flagellin-Induced NF-*κ*B/AP-1 Activity of THP-1

**Figure 1:**
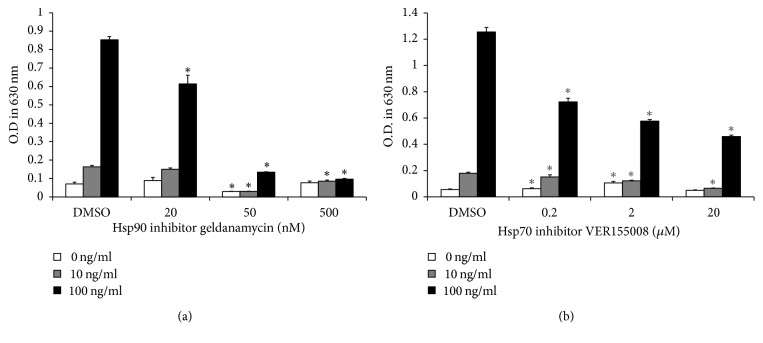
*Inhibition of Hsp90 and Hsp70 decreases flagellin-induced NF-κB/AP-1 activity in THP-1-Xblue cells*. (a) THP-1-Xblue cells were preincubated for 2 h with (a) 20, 50, and 500 nM of the Hsp90 inhibitor GA or (b) 0.2, 2, and 20 *μ*M of the Hsp70 inhibitor VER155008 followed by stimulation with 0 (white), 10 (gray), and 100 ng/ml (black) flagellin from* Salmonella typhimurium* for 24 h. After stimulation, the supernatant was collected and mixed with QUANTI-Blue solution for color development at 37°C. After 2-h color development, absorbance was measured by an ELISA microplate reader at 630 nm. Bar graphs indicate means ± SD (*n* = 6). ^*∗*^*P* < 0.01 versus DMSO-treated group.

To find whether inhibition of Hsp90 may affect flagellin-induced activation of human myeloid leukemia cells, bacterial flagellin-induced NF-*κ*B activation of THP-1 cells in the presence of Hsp90 inhibitor was examined by NF-*κ*B/AP-1 reporting assay. Cells were preincubated with Hsp90 inhibitor geldanamycin (GA) with various concentrations for 2 h, followed by 24 h stimulation with 0, 10, or 100 ng/ml of flagellin. Flagellin-induced NF-*κ*B/AP-1 activity was significantly reduced by the treatment of 50 nM and 500 nM GA compared to the control (Figure 1(a)). Inhibitory effects of 20 nM GA on NF-*κ*B/AP-1 activity were found at the only 100 ng/ml flagellin-treated group (Figure 1(a)). Since it is known that Hsp70 acts as a cochaperone of Hsp90 [26], we also examined the effect of Hsp70 inhibition on flagellin-induced NF-*κ*B/AP-1 activity using Hsp70 inhibitor, VER155008, at the concentrations ranging from 0.2 to 2 *μ*M (Figure 1(b)). Flagellin-induced NF-*κ*B/AP-1 activity was reversed in cells treated with VER155008. However, Hsp70 inhibitor showed less inhibitory potency compared with Hsp90 inhibitor (Figure 1(b)).

**Figure 2:**
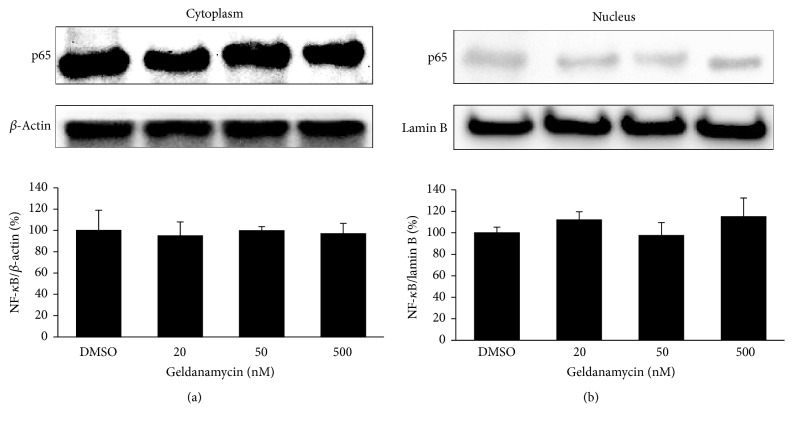
*GA does not affect basal levels of p65 protein in both cytoplasmic and nuclear fractions of THP-1 cells*. Cells were treated with or without the Hsp90 inhibitor GA (20, 50, and 500 nM) for 24 hr. The levels of NF-*κ*B p65 expression in the cytoplasm (a) and nucleus (b) were measured by Western blotting analysis. *β*-Actin was used as a cytoplasm marker and lamin B as a nuclear marker. Data are expressed as means of relative expression ratio ± SD (*n* = 3).

Since previous study has reported that Hsp90 activity is required for I*κ*B kinase biosynthesis and NF-*κ*B activation [27], we examined baseline expression of NF-*κ*B in THP-1 cells treated with various concentrations of GA. As shown in Figure 2, basal expression levels of both cytoplasmic and nuclear NF-*κ*B p65 were not significantly changed by treatment of GA, suggesting that GA treatment does not directly affect baseline levels of NF-*κ*B in THP-1 cells under our experimental conditions.

### 3.2. Hsp90 Inhibitor Enhances TLR5 mRNA Expression, While Reducing Cell Surface TLR5

**Figure 3:**
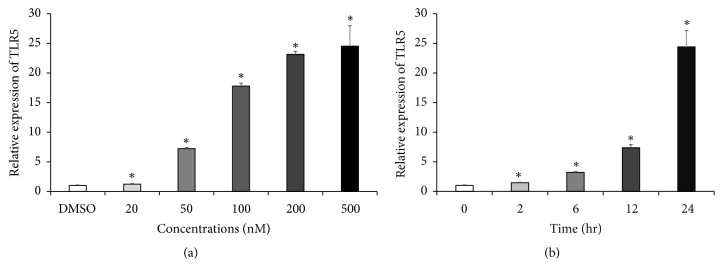
*GA enhances TLR5 mRNA expression in concentration- and time-dependent manners*. Gene expression of TLR5 was determined by qRT-PCR and was normalized to that of *β*-actin. THP-1 cells were treated with or without the Hsp90 inhibitor GA (a) at various concentrations for 24 h or (b) at various time points. Data are expressed as means of relative expression ratio ± SD (*n* = 6). ^*∗*^*P* < 0.05 versus DMSO-treated group.

Next, we examined the effects of various concentrations of GA ranging from 20 nM to 500 nM on TLR5 mRNA expression using a quantitative real-time PCR analysis. TLR5 mRNA expression started to increase with concentration as low as 20 nM and reached its maximal (nearly 25-fold) increase with 500 nM GA (Figure 3(a)). TLR5 mRNA upregulation started 2 h, reaching approximately 25 times the control level at 24 h after treatment with 500 nM GA (Figure 3(b)).

**Figure 4:**
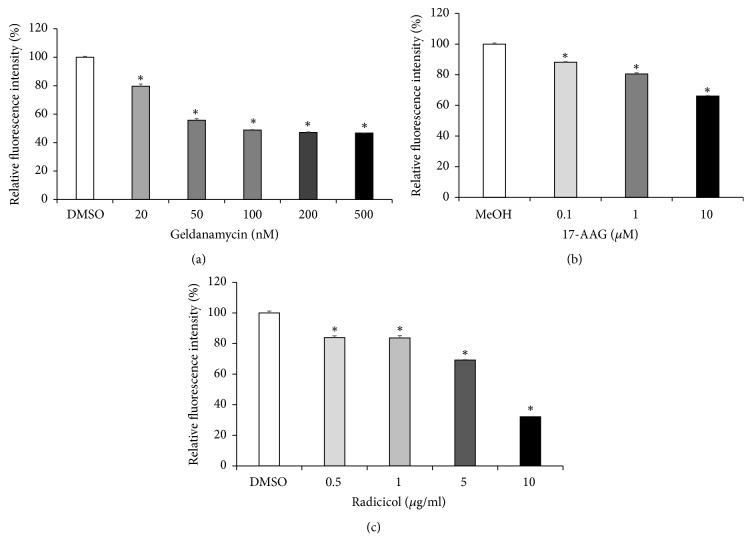
*Inhibition of Hsp90 reduces TLR5 cell surface expression*. Cell surface expression of TLR5 in THP-1 cells was examined by flow cytometry analysis. THP-1 cells were treated with or without the Hsp90 inhibitors, (a) GA, (b) 17-AAG, and (c) radicicol at various concentrations for 24 h. Relative fluorescence intensity was measured, and data are expressed as means of relative expression ratio ± SD (*n* = 6). ^*∗*^*P* < 0.05 versus DMSO- or MeOH-treated group.

In an attempt to elucidate the mechanism by which Hsp90 inhibitor causes the reduction in NF-*κ*B/AP-1 activity induced by flagellin in the THP-1 cells, surface expression levels of TLR5 were evaluated after GA treatment. As shown in Figure 4(a), GA exhibited suppressive effects on surface TLR5 expression in a concentration dependent manner. This effect was further confirmed by other Hsp90 inhibitors, such as 17-AAG and radicicol. Like GA, these inhibitors also caused the reduction in cell surface TLR5 expression in concentration dependent manner (Figures 4(b) and 4(c)).

**Figure 5:**
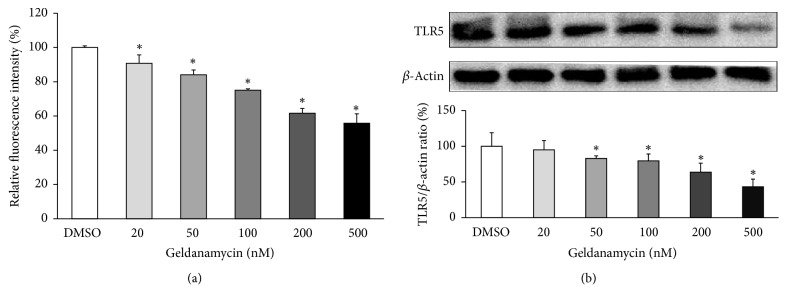
*Inhibition of Hsp90 reduces total protein levels of TLR5*. THP-1 cells were treated with or without the Hsp90 inhibitor GA (20, 50, 100, 200, and 500 nM) for 24 hr. The levels of TLR5 protein expression were measured by intracellular flow cytometry (a) or Western blot (b). Shown are representative blots and densitometric ratios of proteins normalized to *β*-actin. Data are expressed as means of relative expression ratio ± SD (*n* = 3 or 4). ^*∗*^*P* < 0.05 versus DMSO-treated group.

To explain the apparent discrepancy between reduction in TLR5 surface expression and increased mRNA expression, we examined total protein levels of TLR5 of THP-1 cells treated with various concentrations of GA by both flow cytometry and Western blot analysis. Similar to surface TLR5 expressions, total protein levels of TLR5 were significantly decreased by GA treatment in a concentration dependent manner (Figure 5).

### 3.3. Inhibition of Hsp70 Enhances TLR5 mRNA Expression, While Reducing Cell Surface TLR5

**Figure 6:**
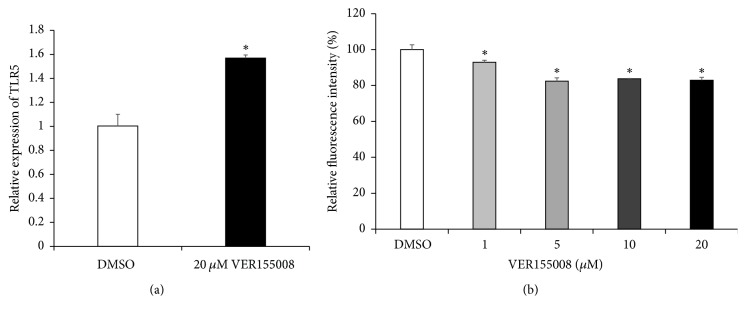
*Inhibition of Hsp70 enhances TLR5 mRNA expression, while reducing TLR5 surface expression*. (a) The Hsp70 inhibitor VER155008 enhances TLR5 mRNA expression in THP-1 cells. Gene expression of TLR5 was determined by qRT-PCR and was normalized to that of *β*-actin. THP-1 cells were treated with or without 20 *μ*M VER155008 for 24 h. Data are expressed as means of relative expression ratio ± SD (*n* = 6). ^*∗*^*P* < 0.05 versus DMSO-treated group. (b) Cell surface expression of TLR5 in THP-1 cells was examined by flow cytometry analysis. THP-1 cells were treated with or without VER155008, at various concentrations, for 24 h. Relative fluorescence intensity was measured, and data are expressed as relative TLR5 expression ± SD (*n* = 6). ^*∗*^*P* < 0.05 versus DMSO-treated group.

Since HSP70 proteins work closely with the HSP90 molecules to maintain the stability and activities of their client proteins [26], we investigated the effects of Hsp70 inhibition on TLR5 expression of THP-1. Cells were treated with or without Hsp70 inhibitor VER155008 and TLR5 mRNA expression was examined. Like GA, Hsp70 inhibitor increased the level of TLR5 mRNA expression (Figure 6(a)), whereas it decreased cell surface TLR5 expression (Figure 6(b)). However, the increase in TLR5 mRNA expression and the reduction in cell surface TLR5 expression by Hsp70 inhibitor were much less than those by Hsp90 inhibitor.

## 4. Discussion

In the present study, we demonstrated that Hsp90 or Hsp70 inhibitors suppress flagellin-induced NF-*κ*B activation in the human myeloid leukemia cell line THP-1. The reduction in cell surface expression of TLR5 caused by Hsp90 or Hsp70 inhibition was found to be responsible for this reduced activity. Although we do not yet know reasons for the perfect negative correlation between mRNA and protein levels of TLR5 seen in the present study, considering the chaperoning function of Hsps, inhibition of Hsp90 or Hsp70 could interfere with proper folding of TLR5, which inhibits movement of mature TLR5 molecules from the endoplasmic reticulum (ER) to the cell surface. In particular, since gp96, an ER paralog of Hsp90 [28], is the master immune chaperone for both cell surface and intracellular TLRs, including TLRs 1, 2, 4, 5, 7, and 9 [29], inhibition of gp96 by GA [30] can cause reduction in TLR5 surface expression. This may explain why Hsp90 inhibitors exerted more potent inhibitory effects than the Hsp70 inhibitor. However, to confirm our speculation that the reduction in the levels of newly synthesized TLR5 proteins is largely due to consequences of ER quality control mechanisms in the presence of Hsp90 inhibitor, it is necessary to examine ER-associated protein degradation, which is a process for detecting and removing misfolded proteins [31], in future study. Regarding the reasons for increased mRNA levels of TLR5, we cautiously speculate that cells treated with Hsp inhibitors may upregulate TLR5 mRNA expression to compensate for the loss of the TLR5 proteins.

Innate immune cells can recognize various tumor-derived antigens through TLRs [32]. Thus, TLR-mediated activation of innate immune cells might play a role in counteracting tumor cells [33]. Indeed, much attention has been recently paid to tumor immunotherapy, which uses TLR agonists to enhance the sensitivity of innate immune cells to tumor-derived antigens [34]. Several TLR agonists have been investigated under preclinical and clinical evaluation [35, 36], and some TLR agonists have been approved for use in tumor therapy [37–41]. However, the current barrier to effective tumor treatment is that the tumor itself is highly immunosuppressive to innate immune responses [42]. Tumor cells and tissues also express TLRs, and elevated expression of TLRs promotes tumor cell survival and proliferation [43, 44]. Furthermore, persistent activation of TLR signaling and the resultant proinflammatory microenvironment facilitate tumor progression and immune suppression by decreasing the cytotoxicity of immune cells, increasing the production of proinflammatory factors, and metastasis [43, 45–47]. Thus, targeting the expression and activation of TLRs in tumor cells might be an important therapeutic strategy to treat tumor. In this regard, our results, showing that treatment with Hsp90 inhibitors induced the suppression of TLR5 cell surface expression and NF-*κ*B activation in THP-1 cells, suggest that Hsp90 inhibitors could be useful to treat tumors, especially human myeloid leukemia that was previously reported to be associated with enhanced TLR5 expression and signaling [24].

The Hsp90 inhibitors used in this study are pan-inhibitors that have limitation to elucidate the role of particular isoforms of Hsp90. Therefore, the use of selective inhibitor of particular isoform of Hsp90 would be necessary to understand the role of each isoform in the modulation of TLR5 expression and flagellin-induced NF-*κ*B/AP-1 activity in future study. In addition, the effects of Hsp70 inhibitors should be more fully explored in future study.

In summary, our results show for the first time that Hsp90 inhibitors suppress TLR5 surface expression and subsequently inhibit NF-*κ*B activation in human myeloid leukemia THP-1 cells. We suggest that these inhibitory effects may be associated with the possible mechanisms by which Hsp90 inhibitors suppress myeloid leukemia.
